# Targeting amino acid metabolism to inhibit gastric cancer progression and promote anti-tumor immunity: a review

**DOI:** 10.3389/fimmu.2025.1508730

**Published:** 2025-02-13

**Authors:** Yuchun Jiang, Qing Tao, Xuehan Qiao, Yufei Yang, Chen Peng, Miao Han, Kebin Dong, Wei Zhang, Min Xu, Deqiang Wang, Wen Zhu, Xiaoqin Li

**Affiliations:** ^1^ Department of Oncology, The Affiliated Hospital of Jiangsu University, Zhenjiang, China; ^2^ Institute of Digestive Diseases, Jiangsu University, Zhenjiang, China

**Keywords:** amino acid metabolism, immunotherapy, chemotherapy resistance, tumor microenvironment, methylation

## Abstract

The incidence of gastric cancer remains high and poses a serious threat to human health. Recent comprehensive investigations into amino acid metabolism and immune system components within the tumor microenvironment have elucidated the functional interactions between tumor cells, immune cells, and amino acid metabolism. This study reviews the characteristics of amino acid metabolism in gastric cancer, with a particular focus on the metabolism of methionine, cysteine, glutamic acid, serine, taurine, and other amino acids. It discusses the relationship between these metabolic processes, tumor development, and the body’s anti-tumor immunity, and analyzes the importance of targeting amino acid metabolism in gastric cancer for chemotherapy and immunotherapy.

## 1 Introduction

Gastric cancer (GC) is a common malignancy of the digestive tract. The early diagnosis rate of gastric cancer is low, with high morbidity and mortality (1). *Due to the lack of early detection biomarkers*, most patients with GC are already at an advanced stage when diagnosed, losing the opportunity for radical surgical resection. Chemotherapy is still an indispensable treatment for gastric cancer, however, gastric cancer patients often develop resistance to chemotherapeutic drugs in the advanced stages, resulting in poor treatment effects and low survival rates for many patients. For example, conventional chemotherapeutic agents, such as cisplatin and 5-fluorouracil, have reduced sensitivity to GC cells, which limits the therapeutic efficacy of treatment (2), highlighting the need for alternative therapies.

The mechanisms of chemoresistance in GC include the activation of DNA repair mechanisms (3), inhibition of cell death pathways (4), and dysregulation of multiple non-coding RNAs (5, 6). For example, activation of the PI3K/AKT/mTOR (7) and RAF/MEK/ERK (8) signaling pathways in paclitaxel-resistant GC cells makes them resistant to chemotherapeutic drugs, and the use of pathway inhibitors can improve the therapeutic effect. p53 cell cycle protein defects can also lead to chemotherapeutic drug resistance in cancer cells (9). GC stem cells (GCSCs) maintain their stemness through the WNT and Hippo pathways, which in turn affects chemosensitivity (10, 11)

*Amino acid metabolism can also affect chemoresistance through different mechanisms, including providing metabolic feedstock, influencing redox homeostasis, and regulating epigenetic modifications. Understanding the role of these metabolic pathways in chemoresistance can help develop new therapeutic strategies to overcome drug resistance in cancer treatment.*

Traditional chemotherapy and targeted therapy have limited efficacy, a high incidence of adverse reactions, and poor patient tolerance; the median survival of patients is mostly less than 1 year. Recently, immunotherapy has brought new therapeutic hope for patients with advanced GC (12).Molecular typing of GC based on high-throughput sequencing and bioinformatics analysis revealed the biological features and clinical prognosis of each subtype. Molecular typing has guided the selection of clinical treatment strategies, for example, *the Cancer Genome Atlas (TCGA) classified GC into EBV-infected, microsatellite-unstable, chromosome-unstable, and genome-stable types* (13, 14). Among them, patients with EBV-infected and microsatellite unstable GC have better responses to immunotherapy (15). Tumor cells as well as immune cells and fibroblasts in the TME influence the efficacy of tumor immunotherapy (16). Most human solid tumors exhibit one of three distinct immune phenotypes: inflammation, exclusion, or desert (17). Several characteristic molecular markers help identify patients for whom immunotherapy is effective, including PD-L1 (18), CPS (19), microsatellite instability (20), TMB (21), deficient mismatch repair, dMMR (22), immune infiltration levels (23), and BRCA2 mutations (24).

Intra-tumor, intra-patient, and inter-patient heterogeneity in GC is a major obstacle to the development of systemic therapeutic agents, and although clinical trials have demonstrated the efficacy of immunotherapy, the majority of patients with gastric cancer are not susceptible to immune checkpoint inhibitor monotherapy; thus, there is an urgent need to find new therapeutic approaches to overcome the resistance of patients with GC to ICI therapy. Temporal and spatial heterogeneity, invasiveness, and prognosis of GC are closely related to the tumor microenvironment (TME). The TME is mainly composed of endothelial cells, lymphocytes, macrophages, myeloid cells, and cancer-associated fibroblasts that are involved in inflammation, cancer immunosuppression, angiogenesis, and metastasis (25, 26). The TME specifically affects the immunotherapeutic efficacy and survival of gastric cancer patients, and activated fibroblasts, endothelial cells, immunosuppressive myeloid cell subpopulations, and regulatory T cells are closely associated with the establishment of an immunosuppressive TME in GC (27, 28). Moreover, IFNγ-activated T cell subsets and HLA-II-expressing macrophages are associated with better treatment outcomes (29). Amino acid metabolism plays an important role in the TME, affecting the growth of tumor cells, the function of immune cells, and the immune status of the entire microenvironment, including the dysregulation of T lymphocyte metabolism and macrophage metabolism. Targeting amino acid metabolism to enhance immunotherapeutic efficacy is an emerging cancer therapeutic strategy that involves the modulation of specific amino acid metabolic pathways to influence tumor cell growth and immune responses.

Amino acid metabolism plays an important role in the TME. *Although amino acids are metabolites that provide energy and carbon for biomass synthesis, they also play a signaling role* (30). Amino acid metabolism is associated with chemoresistance and poor immunotherapy outcomes. Immune checkpoints can suppress anti-tumor immunity through amino acid metabolism. Amino acids are involved in multiple steps of anti-tumor immunity in immune cells, and cancer cells are metabolically reprogrammed to obtain sufficient nutrients to meet high metabolic demands (31). The development of anticancer drugs that target the metabolic vulnerability of cancer cells is a therapeutic strategy for cancer treatment (32, 33).

In this article, we review the influence of amino acid metabolism on tumor development in GC, including methionine, cysteine, glutamic acid, serine, and taurine. In addition, we provide guidelines for targeting amino acid metabolism in GC for chemotherapy resistance and immunotherapy.

## 2 Methionine metabolism

Methionine is an essential amino acid, and S-adenosylmethionine (SAM) produced during methionine metabolism is involved in the regulation of many biomolecules, affecting the biological functions of tumors and immune cells. Intervention with enzymes involved in methionine metabolism or restricting the uptake of methionine is beneficial for limiting the development of tumors.

### 2.1 Effects of methylsulfate metabolism on GC progression

Gastric hepatoid adenocarcinoma (HAS) has higher stemness and methionine cycle activity than conventional GC (34). HAS is a subtype of GC with low morbidity, high mortality, and increased epithelial-mesenchymal transition (EMT) activity. Tumor stem cells (TICs), as tumor-initiating cells, are heterogeneous in their metabolism compared to normal tumor cells, which often leads to the development of chemotherapy resistance and even increases the malignancy of the tumor (35). Methionine starvation and backfill experiments demonstrated that tumor-initiating cells are dependent on methionine metabolism, and its main product, SAM, is the source of nucleic acid methylation and histone methylation methyl group, which is catalyzed by Methionine adenosyltransferase IIA(MAT2A). The authors’ study using the MAT2A inhibitor, FIDAS-5, found that it greatly reduced the levels of SAM and SAH in the tumor-initiating cells, and lowered the levels of histone methylation, which resulted in the tumor clonogenic and tumorigenic abilities of the initiating cells were drastically reduced (35). Gene set enrichment analysis (GSEA) of gene expression data from gastric HAS revealed that samples were enriched in biological processes related to folate binding and glycine metabolism, and that the gene expression levels of MAT2A in this pathway differed significantly between the two groups, and could be a potential drug target in HAS (34).

GSEA of gene expression data from gastric HAS revealed that samples were enriched in biological processes related to folate binding and glycine metabolism and that two genes in this pathway, MAT2A and AHCY (SAHH), showed significant differences in gene expression levels between the two groups and may be drug targets for HAS (36). Thus, the development of NNMT inhibitors may contribute to the prevention of GC.

**Figure 1:**
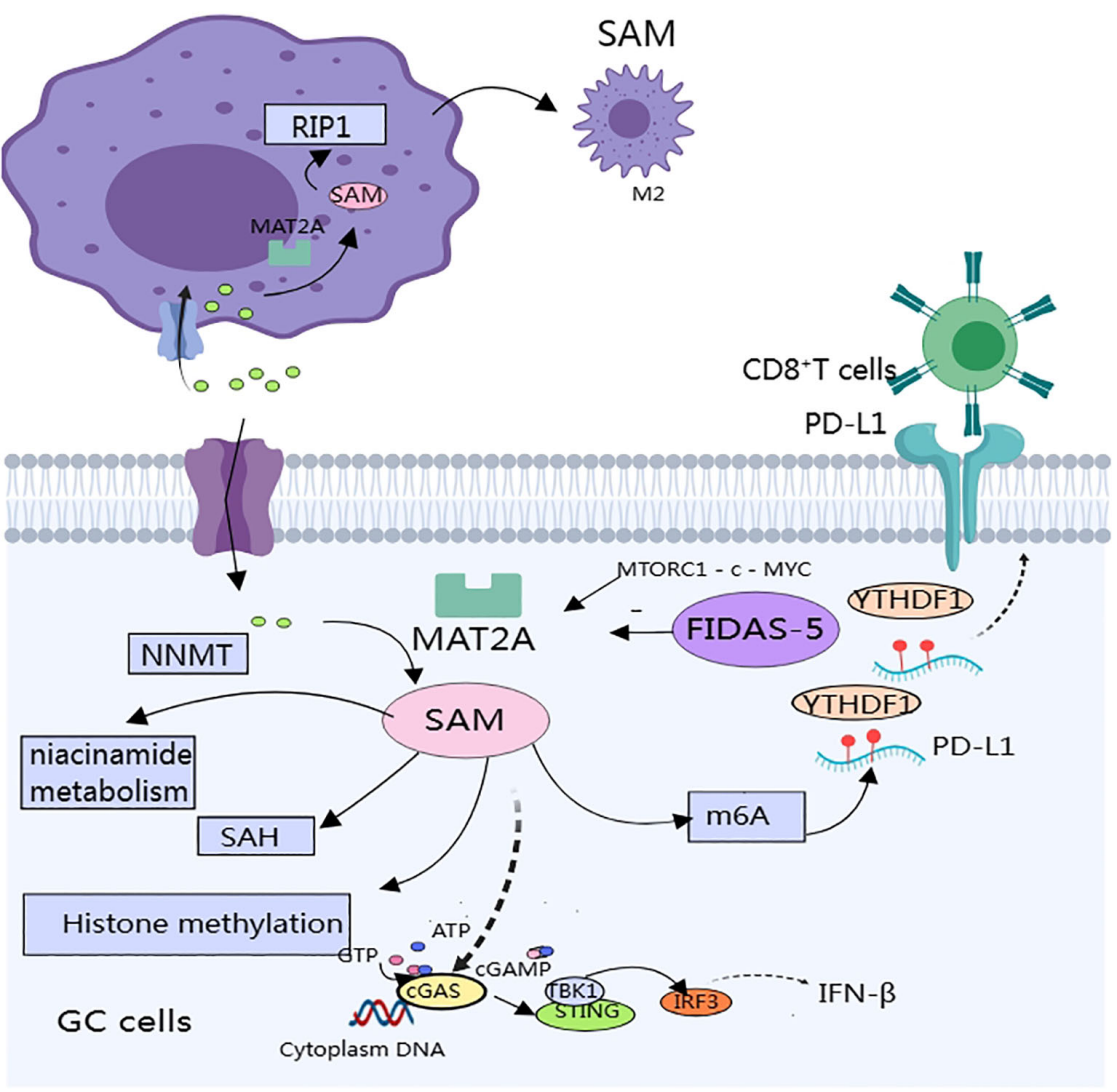
SAM produced by methionine metabolism affects gastric cancer progression and immune components in the tumor microenvironment.

It follows that GC cells may enhance the level of methionine metabolism through the high expression of the key enzyme of methionine metabolism (**Figure 1**), thereby promoting the malignant biological behavior of GC using the methionine metabolite SAM. Therefore, limiting methionine intake or inhibiting a key enzyme involved in methionine metabolism, MAT2A, is a potential therapeutic approach. However, relevant treatments have not yet been popularized in the clinic; most of these studies are preclinical studies conducted in animal models or at the cellular level, and their safety and efficacy in human patients need to be verified by further clinical trials.

### 2.2 Effects of methionine metabolism on antitumor immunity

Methionine metabolism affects multiple aspects of antitumor immunity in gastric cancer: including effects on macrophage polarization, expression of immunosuppressive molecules, and activation of immune pathways (**Figure 1**). Few studies have been published that directly affect the T cells. Targeting the methionine metabolism is expected to enhance the efficacy of immunotherapy and chemotherapy for GC.

*Macrophages play multiple roles in antitumor immunity; they are important components of the immune system and key cell types in the TME* (37). *They recognize and phagocytose pathogens and resist infection while activating other immune cells through the release of signals such as cytokines.* Metabolic interactions between tumor-associated macrophages (TAMs) and malignant cells are critical for maintaining the immunosuppressive nature of the TME.TAMs can be polarized into a pro-inflammatory phenotype M1 and an anti-inflammatory phenotype M2 (38). M1-type TAM mainly exerts antitumor effects, whereas M2-type TAM promotes tumor progression by secreting interleukin (IL)-10 and transforming growth factor-β (TGF-β) (39). In hepatocellular carcinoma, M2 TAMs are the main immune cells that express the immunosuppressive molecule PD-L1 (40). In GC, TAMs trigger regulatory T cells by secreting chemokines, inhibiting the anti-tumor response of T cells, and disrupting immune cell interactions, ultimately leading to the immune escape of GC tumor cells (41). Amino acid metabolism is involved in mediating macrophage polarization, which affects anti-tumor immunity (42), therefore targeting amino acid metabolism to modulate anti-tumor immunity is a promising therapeutic tool.

TAMs in the GC microenvironment predominantly exhibit the M2 phenotype and promote cancer progression through immune escape. Reprogramming of methionine metabolism is tightly linked to the maintenance of the M2 phenotype. TMAs in the GC microenvironment overexpress methionine adenosyltransferase IIA (MAT2A), which increases intracellular levels of SAM and alters the epigenetic pattern of TAMs, *thereby inducing the expression of receptor-interacting protein 1 (RIP1), and the RIP1 upregulation promotes macrophage polarization to M2 type* (43). Therefore, targeting the MAT2A-PIP1 axis is expected to improve the efficacy of immunotherapy.MAT2A-related inhibitors include: substrate-competitive inhibitors and denaturing inhibitors. Only two compounds of this inhibitor have entered clinical studies, and the reasons for the difficulty in applying related inhibitors include poor specificity, difficulty in crossing the blood-brain barrier, and other side effects (44, 45).

SAM produced during methionine metabolism also affects the expression of immune checkpoints in the TME. *In vitro* and *in vivo* experiments have revealed that SAM produced by methionine metabolism promotes the expression of immune checkpoints (PD-L1) and V-domain lg suppressor of T cell activation (VISTA) in tumor cells, which ultimately inhibits T cell activation (46). Specific mechanism: SAM promotes m6A modification of PD-L1 mRNA in tumor cells, and the specific binding protein YTH structural domain family protein 1 (YTHDF1) recognizes and binds to m6A-modified PD-L1 mRNA and promotes PD-L1 expression, methionine dietary restriction or targeting of YTHDF1 enhances the therapeutic effects of PD-L1 blockade (46). In a mouse model of GC, the authors significantly inhibited tumor growth and metastasis by engineering small extracellular vesicles (sEVs) to deliver siRNA targeting m’A reader YTHDF1 (47). Unfortunately, no investigators have yet verified whether inhibition of methionine metabolism has the same effect in GC.

SAM also attenuates the activation of the STING pathway by promoting the methylation of cyclic GMP-AMP synthase (cGAS), resulting in a weakened anti-tumor immune response. cGAS is a genome resistance-associated protein that is associated with genome stability. cGAS is a key molecule of the immune system that is located in the endoplasmic reticulum in the inactivated state and senses cytoplasmic DNA, thereby activating the expression of interferon genes. STING is a key molecule of the immune system, located in the endoplasmic reticulum in the inactivated state, and can sense cytoplasmic DNA to activate interferon gene expression and regulate immune cells to exert anti-tumor effects (48). In a study of intestinal cancer, methionine deprivation promoted the cGAS-sting-interferon pathway and inhibited tumor growth. cGAS is located in both the cytoplasm and the nucleus. cGAS, which is located in the cytoplasm, is the main receptor for cytoplasmic DNA; when stimulated, cGAS produces the second messenger cGAMP, which in turn activates the STING-TBK1-IRF3-INF signaling pathway. Thus, it plays a key role in anti-tumor immunity. SAM methylates cGAS and promotes its cytosolic localization, which decreases its activity. Methionine restriction blocks cGAS methylation, which enhances its cytoplasmic localization and strengthens anti-tumor immunity (49). *In vitro* and *in vivo* experiments have demonstrated that methionine restriction enhances the efficacy of radiotherapy, chemotherapy, and immunotherapy by potentiating the activation of the cGAS-STING pathway. Activation of STING could shift STING to the vicinity of the nucleus and promote macrophage polarization towards pro-inflammatory phenotypes depending on the IL6R - JAK - IL24 pathway, enhance anti-tumor immune responses, and induce apoptosis in GC cells (50). However, no study has linked methionine metabolism to the activation of the STING pathway in GC, and further studies are warranted.

SAM produced by methionine metabolism promotes the malignant biological behavior of tumor cells and inhibits the differentiation and functional roles of immune cells, so targeting MAT2A is expected to be a promising target to promote gastric cancer therapy. However, it has not yet been shown that methionine enters gastric cancer cells and macrophages through which transporter protein specifically, so it is not easy to achieve by restricting transporter proteins to specifically restrict methionine entry into gastric cancer cells or macrophages

## 3 Cysteine metabolism

### 3.1 Effects of cysteine (Cys) metabolism on GC progression

Cys is a semi-essential amino acid (51), and *in vivo* Cys that can be interconverted *in vivo*. Cys is a key component in the synthesis of glutathione (GSH), which is closely associated with the maintenance of a stable cellular redox state.

Glutamate-cysteine ligase (GCL), the rate-limiting enzyme in GSH synthesis, consists of a catalytic subunit (GCLC) and a modifying subunit (GCLM) (52). Experiments in GCLM knockout mice demonstrated that during cancer initiation, cancer cell proliferation and metabolic activity increase, leading to intracellular accumulation of ROS, and that GSH protects cancer cells from oxidative damage by neutralizing reactive oxidants and promoting tumor progression (53). Since Cys is one of the components of GSH, increased production of GSH may deplete the endogenous source of Cys (54). Subsequently, to survive and proliferate, cancer cells accelerate the uptake of extracellular cystine via the CSSC/Glu reverse transporter (xCT transporter) (54, 55). Cysteine enzymes reduce the serum levels of Cys and deplete extracellular Cys. Cysteine enzymes include cysteine proteases, synthetases, and hydrolases and their regulation may be beneficial in controlling cancer progression (56).

**Figure 2:**
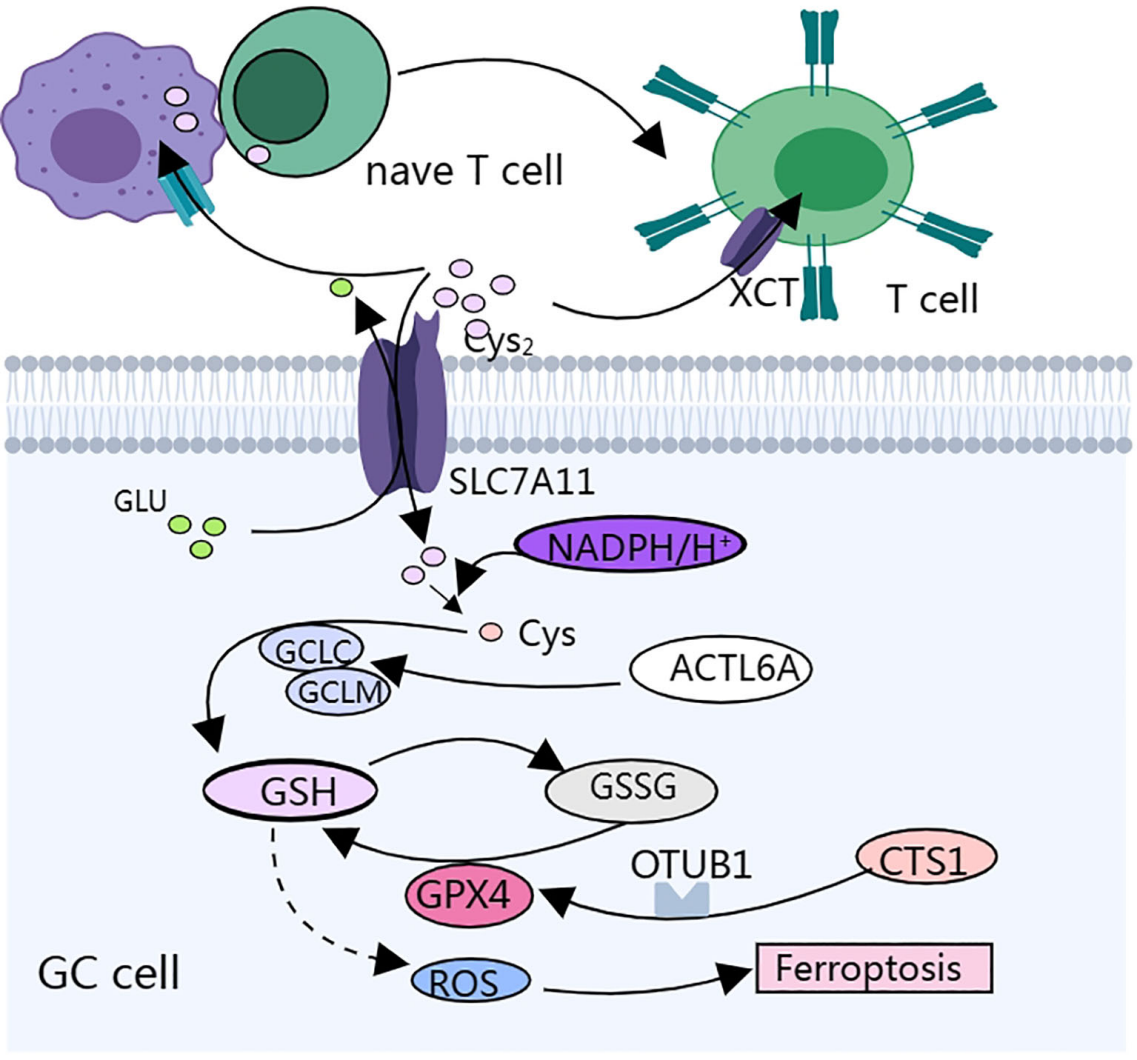
Cysteine metabolism affects gastric cancer progression and the biological function of immune cells mainly by regulating cellular redox status.

Cysteine protease inhibitor (CST1) expression is upregulated in both primary and metastatic GC, suggesting a poor prognosis (**Figure 2**). CST1 can increase the stability of GPX4, a phospholipid hydroperoxide glutathione peroxidase, through two pathways. These include: 1. CST1 interacts directly with GPX4 to increase the stability of GPX4 protein via the proteasome pathway. 2. CST1 also recruits the deubiquitinating enzyme OTUB1 to inhibit GPX4 ubiquitination, which improves the stability of GPX4 and inhibits the expression of ferritin in GC cells. CST1 reduces the intracellular level of ROS by increasing the stability of GPX4, thus inhibiting *ferroptosis* and promoting EMT and metastasis in GC cells (57). The overall efficacy of chemotherapy for metastatic GC is poor; therefore, targeting CST1 may be a promising strategy for the treatment of metastatic GC.

Cystine/glutamate reverse transporter SLC7A11 is a membrane channel transporter protein that can increase the synthesis of L-glutathione (GSH) in GC cells, help cancer cells resist *ferroptosis*-induced reactive oxygen species(ROS)and iron accumulation, enable GC cells to survive under hypoxic conditions, and promote peritoneal metastasis (PM) of GC (58, 59) (**Figure 2**). SLC7A11-mediated cystine uptake promotes GPX4 protein synthesis without affecting GPX4 mRNA or protein degradation (60). Cysteine and methionine deprivation was found to synergize with the GPX4 (glutathione peroxidase 4) inhibitor RSL3 to increase the number of iron-dead cells and lipid peroxidation in mouse and human glioma lines, and a reduced cysteine- and methionine-restricted diet improved the response of mice to RSL3 treatment and prolonged survival (61). GC cells highly express actin-like protein 6A (ACTL6A), which upregulates the expression of the catalytic subunit of γ-L-glutamyl-L-cysteine ligase (GCLC), thereby decreasing the reactive oxygen species (ROS) level and inhibiting *ferroptosis*, and inhibition of ACTL6A and GCLC may be a potential therapeutic strategy for GCs (62).

### 3.2 Effects of cysteine metabolism on tumor immunity

*T lymphocytes are key components of the human immune system and are mainly responsible for regulating the immune response and directly killing infected or tumor cells.* Most effector T cells die through apoptosis after performing their immediate effector function, whereas a small proportion of effector T cells differentiate into memory T cells, which provide a durable protective response to the body (63, 64). T cells in the TME are deprived of amino acids and accumulate metabolites that directly affect their metabolic and immune functions.

Tumor cells overexpressing SLC7A11 compete for cystine uptake, leaving the TME cystine-depleted. Cystine deprivation results in *ferroptosis* and the terminal depletion of CD8+ T cells, resulting in increased cell death and increased PD-1 expression. Knockdown of SLC7A11 in tumor cells, overexpression of SLC7A11 in T cells, overexpression of GCLC in T cells, and cystine supplementation enhanced the anti-tumor ability of CD8+ T cells (65).

Researchers have constructed cysteine gamma-cleaving enzyme (CTH)-overexpressing T cells and used a mouse model of melanoma to perform a metastasis assay of pericytes, a key enzyme in the transsulfuration pathway. CTH-overexpressing T cells have been found to better control tumor growth in mice. The mechanism does not increase cysteine autonomy in T cells but inhibits tumor growth by altering amino acid availability in the tumor microenvironment (66).

Initial T cells lack the cystine/glutamate exchange transporter system, Xct, and therefore rely on neighboring macrophages and dendritic cells (DCs) for cysteine, which is acquired via the alanine-serine-cysteine transporter during the early stages of activation (67, 68). Notably, depleted T cells within the TME typically contain fragmented mitochondria and excess ROS; therefore, the removal of accumulated ROS from the TME is a promising strategy for preventing T cell dysfunction and depletion (69). The antioxidant N-acetylcysteine (N-AC) is a precursor of GSH biosynthesis. In a mouse model, N-AC treatment significantly enhanced the therapeutic effect of over-transferred T cells, which could partially reverse T cell dysfunction by limiting oxidative stress (69).

The TME consists of stromal progenitor cells and tumor-associated mesenchymal stem cells (TC-MSCs). TC-MSCs can play an immunosuppressive role by inhibiting the proliferation of T cells (70), and maturation of dendritic cells (DCs) (71). Cystathionase is a key enzyme for cysteine synthesis. MSCs inhibit cysteine production by suppressing the expression of the cystathionase gene on DCs, which results in T cells lacking cysteine and attenuating T cell immune function (72). Neem Leaf Glycoprotein (NLGP) is a natural glycoprotein extracted from the leaves of neem tree (Neem tree, Azadirachta indica) and is an immunomodulator. IL-10 phosphorylates STAT3 by binding to the receptor on the surface of DCs, and the phosphorylated STAT3 can bind to the promoter region of the cystathionase gene and inhibit its expression. NLGP treatment of TC-MSCs significantly attenuated the expression of IL-10, restored the expression of the cystathionase gene, and promoted the proliferation of T cells and the recovery of their functions (73). However, there have been no studies on the use of NLGP for the treatment of GC.

A subgroup of patients with GC with high expression of human cysteine protease gene (CTSL) and low expression of CD4 lineage transcription factor gene (ZBTB7B) had higher immune infiltration and lower tumor purity, however, this group of patients had the shortest survival and the worst prognosis, and further studies found that this subgroup showed higher levels of immune checkpoints and more abundant cancer-related signaling pathways, including the TGF-β signaling pathway, targeting which may enhance the efficacy of immune checkpoint inhibitors in these patients, and that this subgroup may be able to benefit from the use of TGF-β signaling pathway. This subgroup shows higher levels of immune checkpoints and a richer array of cancer-related signaling pathways, including the TGF-β signaling pathway, and targeting this pathway may enhance the efficacy of immune checkpoint inhibitors in these patients (74). However, this has not yet been experimentally verified.

## 4 Glutamate metabolism

### 4.1 Effects of glutamate metabolism on GC progression

Glutamic acid is a non-essential amino acid that is interconvertible with glutamine, an important metabolic substrate and energy source critical for the growth and survival of many tumor cells (75, 76). Differences in microbial composition and metabolites in proximal and distal GCs were found using RNA sequencing, which may reveal different mechanisms of gastric carcinogenesis and progression; glutamate and glutamine, as microbial metabolites, may be associated with gastric carcinogenesis (77). Analysis of water-soluble metabolites in gastric tissues of a gastric rat model using nuclear magnetic resonance (NMR)-based metabolomics revealed that the metabolic profiles of GC tissues were significantly different from those of normal tissues, which may be related to changes in glutamate metabolism (78). The glutamate metabolic pathway is associated with the immune microenvironment and metabolic remodeling in GC (79).

Cancer cells take up glutamine through glutamine transporter protein (ASCT2), and the glutamine taken up is broken down into glutamate by glutaminase in the cell, which is involved in the synthesis of proteins and nucleotides (76, 80). A proportion of 77% of human GC tissues highly expresses the glutamine transporter protein ASCT2, and 12% highly express glutamine synthetase GS. *In vitro* and *in vivo* experiments have demonstrated that the combined inhibition of ASCT2 and GS can effectively block the glutamine pathway in cancer cells, providing a new potential target for the treatment of gastric cancer (81). Topoisomerase inhibitors such as topotecan (TPT) were found to inhibit glutamine uptake through the downregulation of ASCT2, cause oxidative stress, and induce apoptosis through the mitochondrial pathway in cellular experiments, animal experiments, and sequencing techniques (82).

GDH, a key enzyme in glutamine catabolism, is expressed in the cytoplasmic membrane of gastric cancer cells and inhibits tumor growth through the Notch signaling pathway (83). Glutamate metabolism affects 5-FU resistance in gastric cancer, researchers have constructed 5-FU-resistant cell lines, and metabolic analysis has shown that proline dehydrogenase (PRODH) mRNA expression, which catalyzes the conversion of proline to glutamate and the production of superoxide (84).

Glutamate can also act as a neurotransmitter to influence tumor development and it plays a role in regulating intracellular calcium ion concentration through glutamate receptors (85). Aberrant DNA methylation is one of the major mechanisms by which tumor suppressor genes are silenced in GC, and the glutamate receptor GRIK2 has been identified as a novel epigenetic target (86).

GC is highly heterogeneous and characterized by high levels of autophagy, metabolic disorders, and hypoxia (87, 88). Oncogenic signaling-induced metabolic alterations are critical for cancer development. Activated mTOR regulates cell growth and metabolism. m TOR signaling regulates the three major metabolisms and inhibits catabolic processes such as autophagy (89–91). When nutrients are deficient or subjected to other self-induced signals, mTOR activity is inhibited, and autophagy is initiated (91).

**Figure 3:**
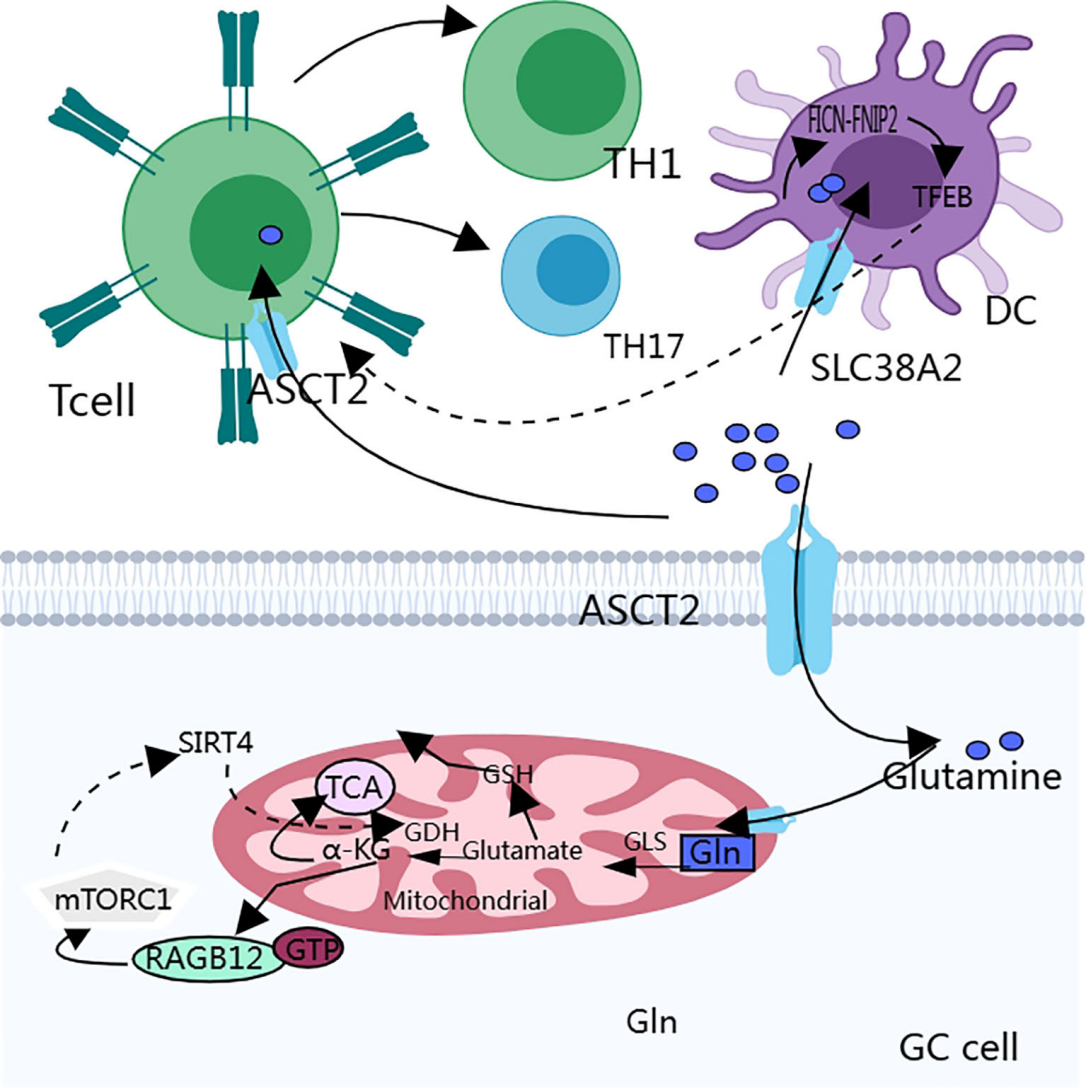
Glutamine metabolism interacts with the autophagy process of cancer cells, which in turn affects the biological behavior of cancer cells. In addition, the anti-tumor immune function of T cells is easily disturbed because they lack glutamine transporters and have a weaker uptake capacity for glutamine than cancer cells.

Autophagy interacts with amino acid metabolism to provide energy to the cancer cells (**Figure 3**). mTORC1 regulates glutamine metabolism, and SIRT4 is a mitochondria-localized member of the sirtuin family, which is a nicotinamide adenine dinucleotide (NAD)-dependent enzyme. sirtuin 4 inhibits the activity of glutamate dehydrogenase (GDH) through ADP-ribosylation, and mTORC1 promotes SIRT4 (92). mTORC2 may promote glutamine catabolism by inhibiting the expression of GS expression (93). Prolonged mTOR inhibition also induces an adaptive increase in glutamine catabolism, allowing tumor cells to avoid the loss of glycolysis and thus mediate resistance to mTOR inhibition (94). Glutamine activates mTORC1 and induces glutamine-dependent cell death (glutamine apoptosis) via two parallel metabolic pathways (95). One: Glutamine is metabolized to α-ketoglutarate (α-KG) via glutaminase (GLS) and glutamate dehydrogenase (GDH), which ultimately activates mTORC1 via proline hydroxylases (PHDs) by facilitating GTP loading of RAGB12 (96). Second, glutamine can indirectly activate mTORC1 by inhibiting AMPK through asparagine synthetase (ASNS) and GABA bypass metabolism to produce ATP.

Our experiments demonstrated that the simultaneous inhibition of ASNS and GLS effectively inhibits mTORC1 (95). Glutamine-mediated apoptosis can be used in combination with existing cancer treatments (e.g., chemotherapy and radiotherapy) to enhance therapeutic effects. For example, drugs that inhibit mTORC1 can be used in combination with nutrient deprivation therapy to effectively kill cancer cells. Some mTORC1 inhibitors such as everolimus and temsirolimus have been approved for clinical use. A variety of glutaminolytic inhibitors are currently in clinical trials, such as the GLS inhibitors BPTES (97) and CB-839 (98).

### 4.2 Effects of glutamate metabolism on antitumor immunity in GC

The influence of glutamate metabolism on GC immunity is multifaceted and involves multiple dimensions of the TME, immune cell activation, and antitumor effects (**Figure 3**).

mTORC1 is also a central metabolic regulator that coordinates environmental and intracellular signals for T cell activation (99). ASCT2 plays an important role in T cell receptor signaling. ASCT2 deficiency leads to reduced TH1 and TH17 cell production and affects inflammatory T cell responses. ASCT2 activates mTORC1 by promoting glutamine uptake, and the activation of mTORC1 also affects TH1 and TH17 cell differentiation (100).

In the TME, immune cells compete with cancer cells for glutamine; initial T cells lack the cystine/glutamate exchange transporter system (67, 68), and depleted T cells have dysregulated metabolic activities, including glutamine catabolism and ROS accumulation (101). Most tumor cells preferentially rely on glutamine metabolism for proliferation, leading to glutamine deficiency in T cells (102), When cancer cells become addicted to glutamine, the function of dendritic cells, which have an antigen-presenting role, is suppressed, and the anti-tumor immune function is dramatically reduced (103).

Folliculin (FICN) is a tumor suppressor protein that plays an important role in a variety of cell types, particularly in the regulation of cell growth, proliferation, and metabolism (104, 105). Transcription Factor EB (TFEB) is a transcription factor that regulates lysosomal biogenesis and functional activity and affects the antigen-presenting capacity of dendritic cells (DCs) (106). DCs compete with cancer cells for glutamine uptake via SLC38A2 Glutamine availability affects the formation of the FICN-FNIP2 complex, which in turn regulates TFEB activity and affects the antigen-presenting function of DCs, which in turn affects CD8+ T cell activation (103, 107). These studies suggest that SLC38A2 and FICN could be used as drug targets to enhance the efficacy of immunotherapy.

In a mouse model of colorectal cancer, high levels of ammonia in the TME inhibited T cell function, leading to T cell depletion and resistance to immunotherapy, and L-ornithine stimulated the urea cycle and glutamine synthesis to achieve *ammonia detoxification* and reactivate the immune response, resulting in a reduction in tumor growth and prolonged survival in a mouse model of the tumor (108). T cell-based cancer immunotherapy can be improved by modulating *ammonia detoxification* mechanisms. For example, by upregulating the expression of CPS1, a key enzyme in the urea cycle, ammonia levels can be reduced, and the survival and anti-tumor effects of CD8+ memory T cells can be improved (109).

Glutamine metabolism can modulate antitumor immunity, but these experiments were not studied simultaneously in the presence of GC. Future studies could be conducted in a mouse model of GC, which could allow observation of the overall impact of the relevant therapeutic agents.

## 5 Serine metabolism

### 5.1 Effects of serine metabolism on GC progression

**Figure 4:**
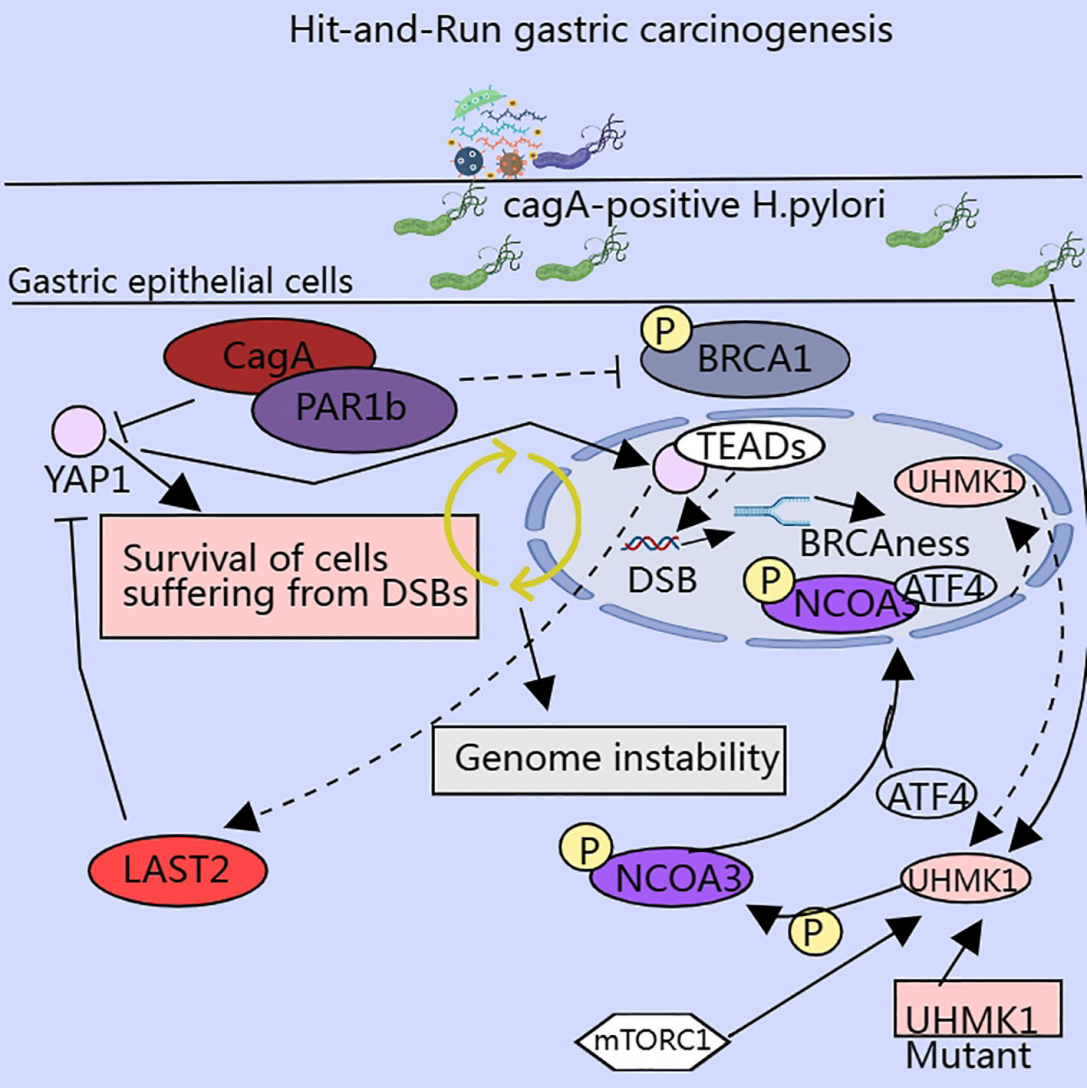
Enzymes involved in serine metabolism influence the malignant potential of cancer cells via multiple pathways.

CagA-positive *Helicobacter pylori* (*H. pylori* CagA) plays a key role in gastric carcinogenesis, and Hippo pathway dysregulation occurs frequently in GC and is involved in *H. pylori*-induced gastric carcinogenesis (110, 111) (**Figure 4**). The Hippo pathway is a serine-threonine kinase cascade reaction that typically consists of two parts. First, a series of serine-threonine kinases are core components of the pathway, such as MST1/2 and LAST1/2. The second is a transcriptional cofactor, Yes-associated protein (YAP), with a transcriptional co-activator and PDZ-binding motif (TAZ) (112, 113). The activation of MST1/2 can activate LAST1/2 by phosphorylation and activated LAST1/2 can phosphorylate and inhibit the activity of YAP and TAZ, thereby inhibiting cell growth and value addition. Dysregulation of this pathway is associated with invasion (114), metastasis (115), chemoresistance (116), and maintenance/expansion of tumor stem cell (CSCs) compartments in malignant tumors (117). CagA invades gastric epithelial cells to form the Cag A/PAR1b complex, which can inhibit polarity-regulated kinase PAR1b-mediated phosphorylation of BRCA1, thereby affecting nuclear translocation of BRCA1, and the lack of nuclear BRCA1 leads to BRCAness, a cellular state that promotes DNA double-strand breaks (DSBs), and the Cag A/PAR1b interaction can also stimulate the Hippo signaling pathway, which promotes the translocation of YAP from the cytoplasm to the nucleus, allowing it to form a complex with the TEAD family, thus exerting an anti-apoptotic effect and giving the cell time to repair DSBs through error-prone mechanisms (110). A study on hepatocellular carcinoma found that the small-molecule inhibitor XMU-MP-1 promoted liver regeneration by inhibiting MST1/2 activation (113).

However, there are not enough studies to show that serine metabolism can directly act on the Hippo pathway. Serine can promote cell growth and proliferation by activating mTORC1, and the activation of YAP/TAZ can promote the transcription of SLC7A5, which promotes the activation of the mTORC1 pathway, thereby exerting a pro-tumorigenic effect (118, 119). This suggests that serine metabolism indirectly regulates the Hippo signaling pathway through the mTOR signaling pathway.

SEM-type gastric carcinomas exhibit marked resistance to chemotherapy, and it was found that these carcinomas highly express the glutaminase GLS, but they are resistant to glutamine catabolism inhibition. Serine availability can affect the catalytic activity of PHGDH and the overall efficiency of the serine synthesis pathway. Under glutamine starvation, SEM-type GC cells maintain cell survival by upregulating the mitochondrial folate cycle pathway driven by 3-phosphoglycerate dehydrogenase (PHGDH), which produces nicotinamide adenine dinucleotide phosphate (NADPH) to scavenge reactive oxygen species (ROS) (120). The therapeutic modality of combined inhibition of GLS and PHGDH has potential clinical applications (120, 121).

SHMT2 plays a key role in serine metabolism, especially in the one-carbon metabolic pathway in mitochondria, affecting methylation and nucleotide synthesis (122). SHMT2 is highly expressed in GC and is associated with poor prognosis (123, 124). *In vitro* and *in vivo* experiments verified that SHMT2 knockdown promotes GC progression through various pathways such as regulating the stability of HIF1α, affecting the downstream VEGF and STAT3 pathways, and maintaining redox homeostasis in GC cells under hypoxic conditions (124).

*Tumor metastasis requires adaptation to new environments.* The lack of serine and glycine in the brain may inhibit tumor cell growth. However, the occurrence of brain metastases suggests that tumor cells can overcome this metabolic limitation. Using proteomics, metabolomics, and multiple brain metastasis models, researchers have demonstrated that PHGDH is a major determinant of brain metastasis in various human cancer types and preclinical models. Enhanced serine synthesis is important for nucleotide production and proliferation of highly aggressive metastatic brain cells. Furthermore, the copy number of the oncogene MYC is high in patients with lung cancer brain metastases, and MYC upregulates the expression of pyridoxal phosphate-binding protein SHMT2, which catalyzes the synthesis of serine, glycine, and nucleotides. The use of PHGDH and SHMT1/2 inhibitors may be beneficial for treating brain metastases (125–127). The incidence of brain metastasis from GC is relatively low. Patients with meningeal metastases from GC have increased MYC copy numbers in their cerebrospinal fluid. This suggests that brain metastases from GC may overcome metabolic limitations via this mechanism (128). Therefore, targeting metabolism is not an effective strategy for cancer treatment. However, it has been demonstrated that inhibiting metabolism can sometimes inhibit the growth of normal cells while inhibiting the growth of cancer cells. Therefore, it is essential to implement a reasonable treatment plan considering specific circumstances.

*UHMK1, a serine and threonine kinase, is highly expressed in GC tissues and promotes GC progression by enhancing the synthesis of slave purines. It was found that UHMK1 enhances the expression of purine metabolism-related genes by phosphorylating Recombinant nuclear receptor co-activator 3 (NCOA3), which strengthens the binding ability of NCOA3 and ATF4. ATF4 has a role independent of the UHMK1-NCOA3-ATF4 axis, and silencing ATF4 also inhibited serine metabolism and glycolysis. H. pylori infection induced NCOA3 - S1062/T1067 phosphorylation and enhanced the promoter activity of UHMK1 via ATF4. The above results suggest that UHMK1 may be an attractive therapeutic target for GC* (129). A study of human fibroblasts demonstrated that TGF-β1 also stimulates ATF4 expression, contingent on the TGF-β1–mTORC1–ATF4 axis, which facilitates serine–glycine synthesis to ensure sufficient amino acids for cell growth and metabolism. Targeting the TGF-β1–mTORC1–ATF4 axis may represent a novel therapeutic strategy (130). Moreover, the transcription factor ATF4 stimulates the transcription of the cell cycle protein Cyclin A1 and accelerates S-phase progression through the formation of a transcriptional complex with PICH and PoI II, thereby contributing to 5-FU chemoresistance (131).

### 5.2 Effects of serine metabolism on tumor immunity in GC

During T cell activation, mitochondrial proteins undergo significant remodeling, and ultimately the mitochondria undergo metabolic reprogramming and enhancement of one-carbon metabolic pathways. If SHMT2 is inhibited in naïve T cells, this ultimately leads to a decrease in both T cell viability and the abundance of antigen-specific T cells *in vivo*, which closely resembles the metabolic characteristics of many cancer cells (132). Regulatory T cells are important in the maintenance of immune homeostasis; serine promotes glutathione production, and glutathione levels affect serine uptake and synthesis. If the integrity of this feedback loop is critical to ensure the normal function of regulatory T cells, disruption of this loop leads to severe autoimmunity with an enhanced anti-tumor response. For example, specific knockdown of glutamate cysteine ligase (Gclc) in mice leads to increased Treg cell serine metabolism, mTOR activation and proliferation, and decreased Treg cell immune suppression. Cysteine ligase (Gclc) knockdown in mice leads to increased serine metabolism in Tregs, increased mTOR activation and proliferation, and decreased the immunosuppressive capacity of Tregs cells (133). GSH is highly abundant in Tregs. GSH limits mTOR activation by regulating serine uptake and synthesis and maintains the normal expression of forkhead box P3 (FoxP3) to maintain the immune function of Tregs. FoxP3 is a transcription factor that is mainly expressed in regulatory T cells (Tregs). Differentiation, development, and maintenance of function (134).

Serine metabolism is critical for the regulation of macrophage polarization, and serine metabolism reduces IGF1 expression by increasing the promoter abundance of S-adenosylmethionine-dependent lysine trimethylation at position 27 of histone H3, which is regulated through the p38-JAK-STAT1 signaling axis, and by restricting the uptake of exogenous serine or by inhibiting the key enzyme of serine metabolism, phosphoglycerol dehydrogenase PHGDH promotes macrophage polarization to a pro-inflammatory M1 phenotype and enhances anti-tumor immunity (135).

Serine plays an important role in the metabolic pathways of GC and may indirectly influence the anti-tumor immune response in gastric cancer by affecting the functional and metabolic status of immune cells. However, further studies are required to clarify the specific mechanisms underlying serine metabolism in GC development and immunotherapy.

## 6 Branched-chain amino acid metabolism

BCAT1 and BCAT2 are common branched-chain amino acid aminotransferases that convert branched-chain amino acids (BCAAs) to branched-chain alpha keto acid and catalyze the conversion of alpha-ketoglutarate to glutamate. Mutations in the codon responsible for encoding BCAT1 have been identified in clinical GC samples, and these mutations result in higher enzymatic activity of BCAT1, which promotes BCAA catabolism and accelerates cell growth motility and tumor development. Rho protein, a Ras-related guanosine triphosphatase, is involved in the formation of stress fibers and focal adhesions and plays a key role in the reorganization of the cytoskeleton. This mutation results in increased Rhoc activity (136). High BCAA levels can upregulate the expression of the glucose transporter molecule Glut1 on the surface of CD8+ T cells via the phosphatidylinositol 3-kinase–AKT–mTORC1–FoxO1 signaling pathway. This results in altered glucose uptake and the downstream metabolic profile of CD8+ T cells. Furthermore, BCAA supplementation enhances the anti-tumor effects of immunotherapy (137).

## 7 Taurine metabolism

Taurine is a semi-essential sulfur-containing β-amino acid that is widely distributed in mammalian tissues and organs, typically in free form. It is not involved in protein synthesis; however, it can be synthesized endogenously or obtained from food. For example, taurine is synthesized from methionine and cysteine in the human liver. *Other organs* can also uptake taurine from the bloodstream. Cysteine sulfinic acid decarboxylase is the rate-limiting enzyme in taurine synthesis. Taurine is formed via the metabolism of methionine and cysteine in hepatocytes. Other cells can also uptake taurine from the bloodstream. Cysteine sulfinic acid decarboxylase has a low activity in the human body, making it susceptible to taurine deficiency. Taurine is also present in the TME and may affect the metabolism of tumor and immune cells (138–141). GC cells *upregulate taurine transporter SLC6A6*, *competing* for taurine in the microenvironment and triggering endoplasmic reticulum (ER) stress in CD8+ T cells to upregulate ATF4. The specific mechanism is that taurine deficiency induces eIF2α phosphorylation, which subsequently promotes the transcription of *ATF4* through PERK–JAK1–STAT3 pathway activation and ultimately leads to the upregulation of immune checkpoint expression and the depletion of CD8+ T cell function. Taurine supplementation improves this state (142). Resistance to chemotherapy is frequently associated with poor therapeutic efficacy. Chemotherapy resistance leads to an increase in the expression of SP1 and thus SLC6A6 in GC cells, which results in a lower level of taurine in the microenvironment. These findings suggest that chemotherapy-induced immunotherapeutic resistance can be improved by targeting the SP1–SLC6A6 axis (142).

## 8 Discussion

GC is the fifth most common malignant tumor worldwide and has an extremely high mortality rate. The reprogramming of amino acid metabolism in GC provides a favorable environment for its growth and survival and can affect the biological functions of both cancer cells and immune cells by participating in multiple signaling pathways through the TME. Therefore, therapeutic strategies targeting amino acid metabolism may provide new options to enhance the effects of chemotherapy and immunotherapy in GC. However, amino acid metabolism is complex, and different amino acid metabolic processes interact to regulate pathway activation. Targeting amino acid metabolism can help regulate anti-tumor immunity and inhibit the progression of GC, but the number of drugs that have entered clinical trials is limited to animal experiments. The reasons for the limitations of drug application in clinics are as follows: 1. Amino acids are not only nutrients for cancer cells but also for many normal cells, and the human body is an organic whole; therefore, how to specifically target specific pathways needs further consideration. 2. Therapeutic resistance. Metabolic pathways interact with each other, and after the application of drugs that target amino acid metabolism for some time, cancer cells may develop resistance to specific amino acid pathways. 3. Most experiments remain at the stage of mouse animal testing, and the short research cycle of hormonal mice may not be able to highlight the efficacy of long-term treatments. 4. Amino acid metabolism in mice may differ from that in humans.

It is often difficult to achieve the desired therapeutic effects with a single treatment. Therefore, drugs targeting amino acid metabolism need to be used in combination with other therapeutic approaches (e.g., chemotherapy, radiotherapy, and immunotherapy) to enhance their therapeutic effect. However, designing an effective combination therapy strategy is complex. Although drugs targeting amino acid metabolism are rarely used clinically, studies have shown that this strategy is a promising therapeutic tool.
